# Synergistic estrogenic effects of *Fusarium* and *Alternaria* mycotoxins in vitro

**DOI:** 10.1007/s00204-016-1795-7

**Published:** 2016-07-11

**Authors:** Katharina Vejdovszky, Kathrin Hahn, Dominik Braun, Benedikt Warth, Doris Marko

**Affiliations:** Department of Food Chemistry and Toxicology, University of Vienna, Waehringer Str. 38, 1090 Vienna, Austria

**Keywords:** Xenoestrogen, Natural toxin, Food safety, Food contaminants, Chemical mixtures

## Abstract

Mycotoxins are toxic secondary metabolites formed by various fungal species that are found as natural contaminants in food. This very heterogeneous group of compounds triggers multiple toxic mechanisms, including endocrine disruptive potential. Current risk assessment of mycotoxins, as for most chemical substances, is based on the effects of single compounds. However, concern on a potential enhancement of risks by interactions of single substances in naturally occurring mixtures has greatly increased recently. In this study, the combinatory effects of three mycoestrogens were investigated in detail. This includes the endocrine disruptors zearalenone (ZEN) and α-zearalenol (α-ZEL) produced by *Fusarium* fungi and alternariol (AOH), a cytotoxic and estrogenic mycotoxin formed by *Alternaria* species. For evaluation of effects, estrogen-dependent activation of alkaline phosphatase (AlP) and cell proliferation were tested in the adenocarcinoma cell line Ishikawa. The estrogenic potential varied among the single substances. Half maximum effect concentrations (EC50) for AlP activation were evaluated for α-ZEL, ZEN and AOH as 37 pM, 562 pM and 995 nM, respectively. All three mycotoxins were found to act as partial agonists. The majority of binary combinations, even at very low concentrations in the case of α-ZEL, showed strong synergism in the AlP assay. These potentiating phenomena of mycotoxin mixtures highlight the urgent need to incorporate combinatory effects into future risk assessment, especially when endocrine disruptors are involved. To the best of our knowledge, this study presents the first investigation on synergistic effects of mycoestrogens.

## Introduction

Mycotoxins are toxic compounds produced as secondary metabolites by molds. Contamination of food crops may occur on the field or post-harvest. This leads to the entrance of mycotoxins into the food chain and consequently to a potential risk for human health (Bennett and Klich 2003). The variety of fungal species capable of producing mycotoxins is vast, leading to an even larger diversity of over 300 secondary fungal metabolites already known to possess toxic properties. Studies on mycotoxin contamination profiles in feed and foodstuff reveal that compounds generally do not occur isolated, but in complex mixtures (Domijan et al. 2005; Ezekiel et al. 2012; Serrano et al. 2013; Shephard et al. 2013; Streit et al. 2013). In addition, these co-contaminations are not limited to mycotoxins produced by one certain fungal genus. Accordingly, co-occurrence of mycotoxins of different genera, like *Fusarium* toxins and *Alternaria* toxins, is evident (Sulyok et al. 2010; Uhlig et al. 2013; Warth et al. 2012). As the variety of naturally occurring compounds may suggest, specific modes of action of mycotoxins and manifestation of their toxic effects in humans and animals are highly diverse. Among many other mechanisms of toxicity, several mycotoxins are known for their mutagenicity and genotoxicity, while others may act on cell membrane permeability, inhibit protein synthesis, or induce inflammatory responses (Gross-Steinmeyer and Eaton 2012; Kamyar et al. 2004; Pestka 2010). Interestingly, some mycotoxins also possess endocrine disruptive potential. The most prominent mycoestrogen is the *Fusarium* toxin zearalenone (ZEN) which is capable of binding and activating both human estrogen receptors, α and β (ER-α, ER-β) due to its structural similarity to the body’s own natural hormone 17-β-estradiol (E2). It is described as a full agonist for ER-α and a mixed agonist–antagonist for ER-β (Kuiper et al. 1998). After absorption, ZEN is partly hydroxylated to α- and β-zearalenol (α-ZEL, β-ZEL), which are subsequently reduced to α- and β-zearalanol (α-ZAL, β-ZAL) and/or conjugated with glucuronic acid during phase II metabolism (Frizzell et al. 2015; Warth et al. 2013). Studies on the estrogenic activity of ZEN, and its metabolites, revealed that α-ZEL possesses even stronger estrogenic properties than ZEN itself, whereas β-ZEL is less active (Frizzell et al. 2011; Hagler et al. 1979; Metzler et al. 2010). Due to these effects, ZEN induces estrogenic conditions, especially in swine, which initially resulted in the discovery of this toxin (Coe et al. 1992; McErlean 1952; Stob et al. 1962). Several studies postulate endocrine disruptive potential also in humans. ZEN is known to mediate proliferative effects on estrogen-dependent cancer cells of the breast or the endometrium (Li et al. 2012a). Recent epidemiological studies associate ZEN with perturbed breast development and increased breast cancer risk; however, these studies concentrated on the evaluation of biomarkers in urine which only reflects short-term exposure (Bandera et al. 2011; Belhassen et al. 2015). Apart from its estrogenic activity, ZEN also holds DNA damaging properties and induces oxidative stress (Abid-Essefi et al. 2004; Gao et al. 2013). These effects are supposedly causally connected to cytotoxic effects and are diminished by hydroxylation to α-ZEL or β-ZEL (Abid-Essefi et al. 2004, 2009). Alternariol (AOH), produced by fungi of the genus *Alternaria*, is another mycotoxin capable of inducing estrogenic stimuli. However, this effect of AOH is predominantly mediated via ER-β, which is bound with approximately ten-fold higher affinity than ER-α (Frizzell et al. 2013; Lehmann et al. 2006). Similar to ZEN, AOH possesses cytotoxic potential and has been found to induce oxidative stress and DNA damage (Fehr et al. 2009; Tiessen et al. 2013). In vitro estrogenicity and cytotoxicity are, in some measure, quite contrary as estrogenic effects often involve growth stimuli. Supposable criteria to be decisive for the dominant impact are primarily the amount of mycotoxin, but also the duration of exposure. *In vivo*, predominant effects may additionally be influenced by specificities of target organ tissues concerning available estrogen receptors and metabolizing enzymes.

Recently developed innovative LC–MS/MS-based multi-toxin methods revealed the common occurrence of *Fusarium* and *Alternaria* toxins in food and feed (Sulyok et al. 2010; Uhlig et al. 2013; Warth et al. 2012). Therefore, the importance to evaluate combinatory effects of compounds produced by these genera is evident, as humans and animals are constantly exposed to co-contaminated diets. Any kind of effect inherent to a mycotoxin, like cytotoxicity, genotoxicity or estrogenicity, may potentially be influenced by interactions with other mycotoxins. Currently, insufficient research effort is put into the elucidation of mixture-effects involving endocrine disruptive mycotoxins despite its great importance regarding consumer’s risk.

The experimental design of this study aims to give a detailed profile on the combinatory estrogenic effects of the mycotoxin ZEN, its most potent estrogenic metabolite α-ZEL, as well as AOH. Wide concentration ranges were tested, in order to cover realistic in vivo exposure scenarios of low levels in the body, as well as rare conditions of high exposure. Massart et al. (2008) reported mean serum levels of ZEN and α-ZEL in girls with precocious puberty of about 3 nM and 300 pM, respectively. Unfortunately, specific serum levels of AOH have not been investigated so far. However, mean AOH concentrations of about 50 nM were found in bakery products, but also much higher levels of about 97 nM in tomato products, 151 nM in sunflower seeds and 380 nM in wheat flour were detected (Hickert et al. 2016; Zhao et al. 2015). Here, several magnitudes below and above these serum levels or concentrations found in foodstuff were tested. Well-established tests on estrogenic stimuli and proliferation were conducted under equal testing conditions, for the detection of transcriptional response and cell growth induction, but also to monitor possible cytotoxic effects. Our study presents first insights into combinatory estrogenic effects of mycotoxins.

## Materials and methods

### Chemicals and reagents

**Fig. 1:**
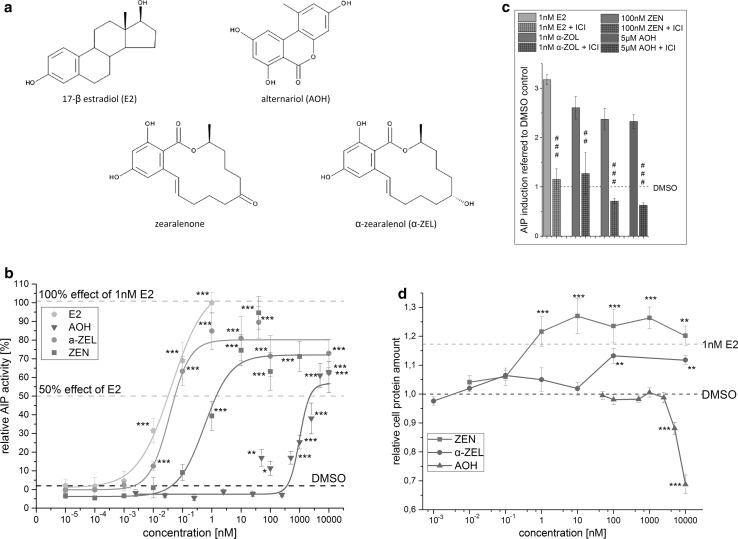
E2 and selected mycotoxins: estrogenic activity in the alkaline phosphatase (AlP) assay and impact on the growth of Ishikawa cells. **a** Chemical structures. **b** Dose–response curves in the AlP assay (mean, SEM). **c** Inhibition of estrogenic effects in the AlP assay by co-incubation with the ER receptor antagonist ICI 182,780 (mean, SEM). **d** Impact on cellular protein amount determined by the SRB assay (mean, SEM). Significant differences to the solvent control (DMSO), or significant differences of co-incubations with ICI 182,780 to single tested substances are indicated by *asterisks*. All measurements were repeated in at least three independent biological replicates, each measured in technical triplicates. (*p > 0.05; **p > 0.01;***p > 0.001)

Cell culture media and supplements were purchased from GIBCO Invitrogen (Karlsruhe, Germany). Alternariol, zearalenone, α-zearalenol, 17-β-estradiol, 4-nitrophenylphosphate, diethanolamine and MgCl_2_ were purchased from Sigma-Aldrich (Schnelldorf, Germany). DMSO, Triton X-100 and Tris were purchased from Roth (Karlsruhe, Germany). Trichloroacetic acid was purchased from VWR (Radnor, PA, USA). ICI 182,780 was purchased from Tocris (Bristol, United Kingdom). Chemical structures of the tested substances are shown in Fig. 1a.

### Experimental design

The aim of this in vitro study was to investigate estrogenic effects of combinations of AOH with ZEN or its metabolite α-ZEL. The estrogen-sensitive human endometrial adenocarcinoma cell line, Ishikawa, is a well-suited model system for the detection of estrogenic stimuli (Holinka et al. 1986; Johnson et al. 2007; Lehmann et al. 2006) and was chosen as model system for this study. Two assays were used to evaluate estrogenic properties. Transcriptional response to estrogens was evaluated by assessment of alkaline phosphatase activity, encoded by a gene, which is transcriptionally activated upon estrogen receptor activation. The sulforhodamine B assay (SRB) assay was performed to investigate a potential impact on cell growth and at high concentrations of mycotoxins, accordingly, cytotoxic effects. Incubation conditions of both assays were kept equal, to enable a direct comparison of the obtained results.

Preliminary measurements of concentration ranges of the natural estrogen hormone E2 as well as AOH, ZEN and α-ZEL were taken in the AlP assay. Due to the low solubility of AOH, a final concentration of 1 % DMSO in the incubation solution needed to be applied. For details on measured concentrations, see Fig. 1b. The most potent concentration of E2, 1 nM, was always tested in parallel and served as positive control for estrogenic stimuli. Measurements of 1 % of the solvent, dimethyl sulfoxide (DMSO), were taken as solvent control. To confirm that effects arise from estrogenic stimuli, E2 and most potent concentrations of single substances and combinations were co-incubated with 1 µM of the high-affinity estrogen receptor antagonist ICI 182,780. Inhibition of effects by adding ICI 182,780 indicates the involvement of estrogen receptors. Sigmoidal dose–response curve fitting with Levenberg–Marquardt algorithm was applied on data of each single substance. EC50 values and concentrations of single substances reaching the half maximum effect of E2 were extracted from the dose–response curves.

In order to ensure comparability of the data, measurements of combinations were always taken in parallel to measurements of respective single substances at exactly the same concentrations. To ensure a detailed elucidation of combinatory effects between ZEN and AOH or α-ZEL and AOH, all concentrations which showed effects as single substances (ZEN 10 pM–10 µM; α-ZEL 1 pM–10 µM; AOH 50 nM–10 µM) were combined with each other in binary mixtures. In addition, combinations in constant concentration ratios were measured in order to elucidate whether dose–response curves of ZEN and α-ZEL are shifted when combined with AOH. Based on the preliminary measurements, the ratio of 1:250 (ZEN/α-ZEL/AOH), which gave highly potent effects in both combinations, was chosen for these evaluations. Here, measured concentrations ranged for ZEN and α-ZEL from 10 fM to 40 nM and for AOH from 2.5 pM to 10 µM.

### Cell culture

The human endometrial adenocarcinoma cell line Ishikawa was purchased from ECACC (Wiltshire, United Kingdom). Cells were cultivated in humidified incubators (37 °C, 5 % CO_2_) in phenol red free Dulbecco’s Modified Eagle’s Medium containing F-12 Nutrient Mixture (DMEM/F-12) and 10 % FBS (Fetal Bovine Serum). For experiments, FBS was exchanged by charcoal-dextran stripped FBS, which provides low levels of hormones. For all assays, 24 h previous to incubation, Ishikawa cells were seeded at a density of 15,000 cells per well into a 96 well plate. Incubation of AOH, ZEN, α-ZEL and all tested combinations was conducted for 48 h, at a final solvent concentration of 1 % DMSO. Details on tested concentrations are indicated in all figures. All measurements were taken at minimum in three independent biological replicates, each measured in technical triplicates.

### Alkaline phosphatase assay

This assay was conducted as previously described (Lehmann et al. 2006) and optimized. After 48 h of incubation, cells were washed three times with PBS, before cell lysis was initiated by keeping the cells at −80 °C for 20 min. Subsequently, the cell lysate was kept at room temperature for 5 min. 50 µl AlP buffer (5 mM 4-nitrophenylphosphate, 1 M diethanolamine, 0.24 mM MgCl_2_, pH 9.8) was added to each well and was incubated for 5 min. Measurements of absorbance were taken at 405 nm, every 2 min, for 1 h, at 37 °C, with the Cytation 3 Cell Imaging Multi-Mode Reader from Biotek^®^ (Winooski, Vermont, USA). The activity of the alkaline phosphatase was calculated as the slope of the curve, obtained by the measurements monitored over 1 h. Final results were referred to the solvent control.

### SRB assay

This assay was conducted according to Skehan et al. (1990). After 48 h of incubation, 10 µl of 50 % TCA (trichloroacetic acid) was added to each well and incubated for 1 h, at 4 °C, in the dark, to facilitate the fixation of cells. Subsequently, cells were washed four times with H_2_O. The plate was dried at room temperature in the dark, prior to the addition of 50 µl of SRB reagent (0.4 % mass concentration SRB in 1 % acetic acid solution) per well, and further incubation for 1 h at room temperature in the dark. Afterward, wells were washed twice with H_2_O and twice with 1 % acetic acid solution, before the plate was dried at room temperature in the dark. Finally, 100 µl, 10 mM Tris solution (pH 10) was added, incubated for 5 min, in the dark, and absorbance was measured at 570 nm, using the Victor V3 1240 Multilable Counter from Perkin Elmer (Waltham, Massachusetts, USA). Final results were referred to the solvent control.

### Evaluation of combinatory effects in an estrogenic system

Combinatory effects were determined as described previously by Vejdovszky et al. (2016) with minor adaptions to measurements of estrogenic stimuli.

#### Combination index theorem

For CI evaluation, effects were calculated by the following formula, where max is the chosen maximum value of 3.7, *y*_C_ is the measurement of the DMSO solvent control, to which all data are referred to, and *x*_T_ is the measured value of the tested substance or combination.

$${\text{effect}} = \frac{1}{\hbox{max} }*\left( { \frac{{x_{\text{T}} }}{{y_{\text{C}} }}} \right)$$

According to Chou et al. (1975, 1984), measured effects of combinations were evaluated for synergism, antagonism or additivity, based on the *medium effect equation* and the *combination index theorem* (Chou 1975; Chou and Talalay 1984). The equation below specifies the calculation of the combination index (CI). *D*_*1*_ and *D*_*2*_ represent applied mycotoxin concentrations in the combination. *D*m and *m* are two parameters determined via the medium effect equation and describe the potency and the shape of the dose–response curves of each mycotoxin. The actual effect of the combination is termed *fa*.

$$CI = \frac{{D_{1} }}{{D{\text{m}}_{1} \left[ {fa/1 - fa} \right]^{1/m1} }} + \frac{{D_{2} }}{{D{\text{m}}_{2} \left[ {fa/1 - fa} \right]^{1/m2} }}$$

A *combination index* (CI) value of one indicates additive effects, whereas a CI < 1 indicates synergism, and CI > 1 antagonism. A detailed classification of CI values was applied according to Chou (2006) and is illustrated in Fig. 3. As suggested by Chou (2006), the most exact evaluation of combinatory effects is achieved by measuring combinations in constant ratios, which allows comparing dose–response relations of the combination with the single substances based on the *medium effect equation*. CI values can thereby be calculated for the whole effect range of the combination and graphically displayed in the effect-CI plot.

### Statistics

All measurements of combinations, in the AlP assay and the SRB assay, were taken in at least three biological replicates, each in technical triplicates. The whole dose range of respective single substance was always measured in parallel. Significance levels were set to 5 % (^#^, *p > 0.05; ^##^, **p > 0.01; ^###^, ***p > 0.001). All data were tested for normality by Kolmogorov–Smirnov test. Significant differences of all measurements, compared to the solvent control, or to the respective ZEN or α-ZEL concentration effect, were evaluated via one-way ANOVA and Bonferroni post hoc test. For statistical comparison of all measurements to 1 nM E2 with and without ICI 182,780, Student’s *t* test was applied. Statistical analyses were performed with OriginPro 9.1 G (Origin Lab, Massachusetts).

## Results

### Estrogenic stimuli: activation of alkaline phosphatase

Estrogenic effects of single mycotoxins on Ishikawa cells (human endometrial adenocarcinoma) were evaluated after 48 h of exposure, by measuring AlP activity. Sigmoidal dose–response curve fitting was performed for each single substance. By this analysis, the effective concentration that induces 50 % response (EC50), a commonly used measure of toxin potency, can be determined. EC50 values for E2, α-ZEL, ZEN and AOH were calculated as 41, 37, 562 pM and 995 nM, respectively (Fig. 1b). However, these EC50 values are related to the respective maximum effect level of each single substance which indeed vary, as it is apparent in Fig. 1b. A direct comparison of EC50 values may therefore not be very meaningful. To enable a more reasonable comparison of the curves, concentrations of each single substance which reach 50 % of the effect of E2 were extracted from the dose–response curves. Table 1 lists these concentrations in comparison with the EC50 values. To confirm that the observed effects indeed arose from estrogenic stimuli, most potent concentrations of all substances were co-incubated with the high-affinity estrogen receptor antagonist ICI 182,780. Corresponding results, shown in Fig. 1c, demonstrated that the effects of all substances can be suppressed by ICI 182,780 and are therefore considered to depend on estrogen receptor activation.

**Table 1:** EC50 values and concentrations of single substances reaching 50 % of the effect of E2

	EC50^a^	50 % effect of E2^b^
AOH	995 nM	2.251 nM
α-ZEL	37 pM	55 pM
ZEN	562 pM	1.423 nM

^a^Concentration that gives 50 % of the maximum effect level of each single substance

^b^Concentration that gives 50 % of the effect of 1 nM E2

**Fig. 2:**
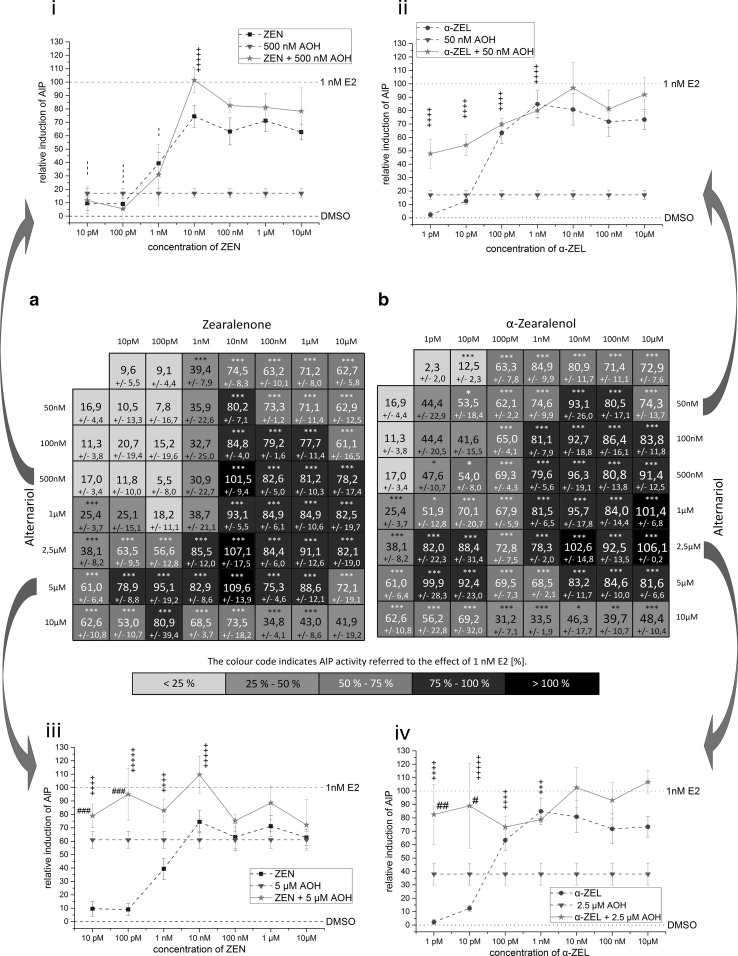
Heatmaps showing relative effects on AlP activity of Ishikawa cells by single mycotoxins and binary combinations of AOH with **a** ZEN or **b** α-ZEL (mean, SEM). Effects of solvent control and 1 nM E2 were set to 0 and 100 %, respectively.* Four line graphs* covering whole concentration ranges of ZEN or α-ZEL in combination with one low (i, ii) and one high (iii, iv) concentration of AOH are extracted exemplary from the data set for comparison with the effects of single substances. Significant differences of effects to the solvent control are indicated by* asterisks*, significant differences of the binary mixtures to measurements with ZEN or α-ZEL, as single compounds, are indicated by* hash marks* (*, ^#^ *p* > 0.05; **, ^##^ *p* > 0.01;***, ^###^ *p* > 0.001). CI values are indicated by + or −, and reflect categorization of interactions according to Chou (2006). All measurements were repeated in at least three independent biological replicates, each measured in technical triplicates

The maximum induction of AlP activity, measured in this experimental setup, was not reached by any single substance, not even by the human hormone E2. At its most potent concentration of 1 nM, E2 reached a mean induction of 3.2-fold compared to the solvent control. This value was defined as 100 %. The maximum induction of AlP activity in this system was measured in combination tests of 10 nM ZEN and 5 µM AOH, which was on average 3.4-fold higher compared to the solvent control. Several other combinations of ZEN and AOH, but also of α-ZEL and AOH, showed effects which also exceeded the effect of 1 nM E2 (see Fig. 2i–iv) . However, these findings were not statistically significant to measurements of 1 nM E2 (*p* < 0.05). All measured effects of single substances and combinations are reported in the heatmaps A (ZEN) and B (α-ZEL) in Fig. 2. Results are expressed as percentage of induction, where 0 and 100 % represent the values of the solvent control and of 1 nM E2, respectively. Significant AlP activation, compared to solvent control, is indicated by asterisks. The color code of these heatmaps indicates the strength of the effect, which enables a visual interpretation of results of all tested combinations. AOH was found to increase the estrogenic effects of both *Fusarium* toxins in almost all tested concentrations. Only AOH in concentrations below 2.5 µM in combination with ZEN below10 nM did not increase AlP activity significantly. Some combinations of α-ZEL below 100 pM and AOH below 1 µM were found to show significant induction of AlP activity. Both data sets, the combinations of AOH with either ZEN or α-ZEL, showed similar trends. Effects were most pronounced at somewhat medium concentrations, but decrease at very high doses. Concentrations of 0.5–2.5 µM of AOH were found to mediate the most potent effects on the estrogenic activity of ZEN and α-ZEL.

In order to visualize the impact of AOH on the estrogenic effects of ZEN and α-ZEL, four exemplary graphs were drawn out of the data set. For each *Fusarium* compound, two line graphs are plotted, showing effects of combinations with AOH, in one low (Fig. 2i, ii) and one high concentration (Fig. 2iii, iv), in comparison with the effects of the single substances.

**Fig. 3:**
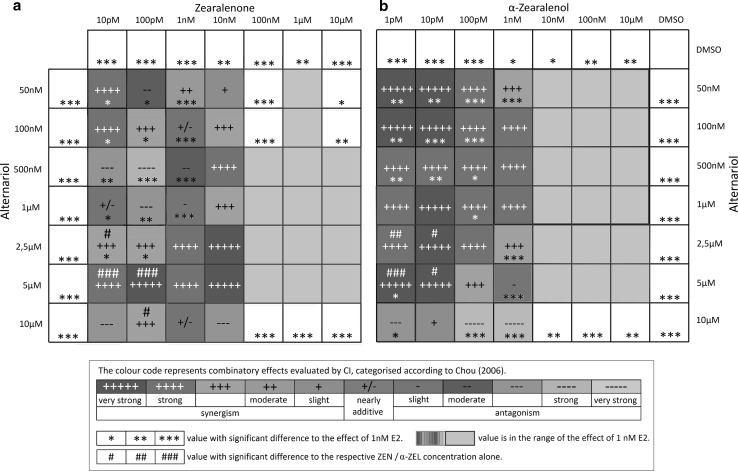
Heatmaps depicting combinatory effects of binary mixtures of AOH with **a** ZEN or **b** α-ZEL calculated on basis of AlP activity. The *color code* represents combinatory effects evaluated by the CI and are categorized according to Chou (2006). Significant differences of effects to the solvent control are indicated by *asterisks*, significant differences of the binary mixtures to measurements with ZEN or α-ZEL, as single compounds, are indicated by *hash marks* (*, ^#^ *p* > 0.05; **, ^##^ *p* > 0.01;***, ^###^ *p* > 0.001). Combinations with nonsignificant differences to the effect of 1 nM E2 are highlighted in *gray* and/or framed in *blue*. All measurements were repeated in at least three independent biological replicates, each measured in technical triplicates (colour figure online)

Combinatory effects of AOH and low concentrations of ZEN or α-ZEL (ZEN: concentrations below 100 nM; α-ZEL: concentrations below 10 nM) were calculated via the *combinatory index* (CI) and categorized according to Chou (2006), regarding synergism or antagonism. To enable correct CI calculations, the maximum value for AlP activity was set slightly above the maximum measured value (3.4 fold induction) compared to the solvent control. Due to the special characteristics of dose–response curves of all three mycotoxins (discussed below), CI calculations of combinations of high concentrations were not applicable. Figure 3 illustrates combinatory effects with CI values indicated by the color code. Significant differences between values of combinations to the respective ZEN or α-ZEL concentration tested alone are indicated by hash marks. In addition, Fig. 3 shows significant differences of single substances and binary combinations, compared to the effect of 1 nM E2, indicated by asterisks. Some combinations showed higher effects than E2, while all tested concentrations of single substances exhibited significant lower effects.

**Fig. 4:**
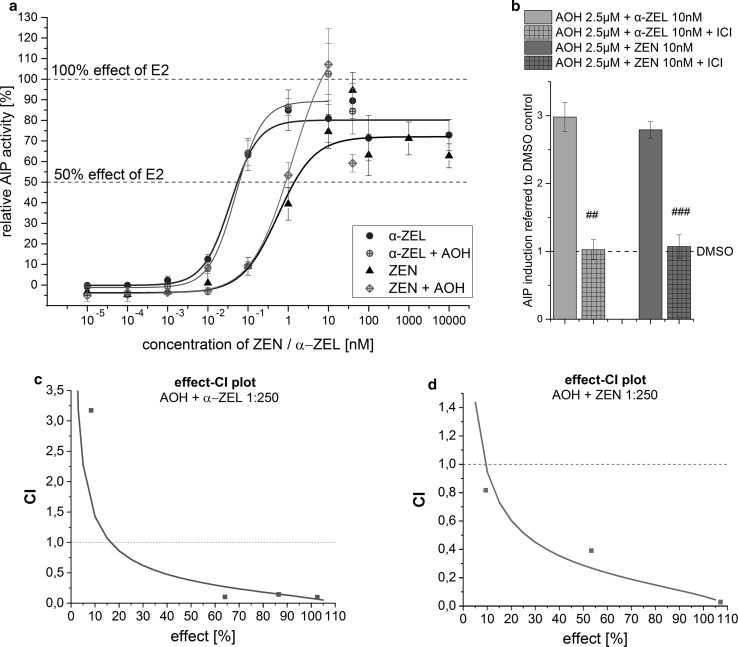
Measurements of combinations in constant ratios of 1:250 (ZEN or α-ZEL:AOH) in the AlP assay. **a** Sigmoidal dose–response curve fits of combinations ZEN + AOH and α-ZEL + AOH in comparison to dose–response curves of ZEN and α-ZEL alone (mean, SEM). **b** Inhibition of estrogenic effects of highly potent combinations (10 nM ZEN or 10 nM α-ZEL + 2.5 µM AOH) in the AlP assay by co-incubation with the ER receptor antagonist ICI 182,780 (mean, SEM). Combinatory index (CI) evaluation of constant ratio combinations 1:250 of **c** α-ZEL + AOH or **d** ZEN + AOH are represented in effect-CI plots (CI < 1 indicates synergism; CI = ~1 indicates additive effects; CI > 1 indicates antagonism): *Dots* display CI values of measured combinations (**a**), *lines* display calculated CI values based on medium effect equation (MEE) analysis of the dose–response curve of combinations

Low concentrations of ZEN (up to 1 nM) in combinations with AOH, at concentrations below 2.5 µM, exhibited some antagonistic effects. However, most combinations from 1 nM ZEN with AOH were found to have synergistic effects. Synergism was also mediated by the majority of higher concentrated mixtures of AOH and ZEN. A very broad concentration range of α-ZEL (1 pM–1 nM) was found to have highly synergistic effects in combination with AOH (see Fig. 3). In both data sets, the majority of combinations tested, especially in high doses, were not found to be significantly different to the effect of 1 nM E2, thus mediating substantial estrogenic effects in the range of E2. As discussed below, this gives evidence for synergism. To verify whether the very high effects of combinations which reach the effect level of 1 nM E2 depend on ER signaling, the highly potent combinations (10 nM ZEN or α-ZEL + 2.5 µM AOH) were co-incubated with the high-affinity ER inhibitor ICI 182,780. This co-incubation leads to complete inhibition of the effects detected for the combinations, which demonstrates their ER dependence (Fig. 4b) .

Results on measurements of constant ratio combinations between ZEN or α-ZEL and AOH (1:250) are shown in Fig. 4. Sigmoidal dose–response curve fitting was performed for the combinations. According EC50 values and concentrations giving 50 % of the effect of E2 are listed in Table 2. Evaluation of CI values over the whole effect range is presented in the effect-CI plots in Fig. 4c, d and indicates mainly synergistic effects between ZEN or α-ZEL and AOH. Only at very low concentrations (10 pM + 2.5 nM), combinations of α-ZEL and AOH were found to mediate antagonism. Similar effects were extrapolated for combinations in very low concentrations of ZEN and AOH based on the effect-CI curve.

**Table 2:** EC50 values and concentrations of constant ratio combinations reaching 50 % of the effect of E2

	EC50^a^		50 % effect of E2^b^	
	ZEN/α-ZEL	AOH	ZEN/α-ZEL	AOH
α-ZEL + AOH 1 : 250	50 pM	12.5 nM	55 pM	13.75 nM
ZEN + AOH 1 : 250	1.241 nM	310.25 nM	865 pM	216.25 nM

^a^Concentration that gives 50 % of the maximum effect level of each single substance

^b^Concentration that gives 50 % of the effect of 1 nM E2

### Impact on cell growth and cytotoxicity

In order to supplement the data on alkaline phosphatase stimuli, mycotoxins and binary combinations were tested in the SRB assay to assess potential growth stimulatory or cytotoxic effects. All experimental conditions were kept equal to the AlP activity measurements to enable direct comparison of the data sets. Effects of single substances in the SRB assay are illustrated in Fig. 1d. ZEN and α-ZEL showed cell proliferating effects increasing with concentration up to 10 µM, whereas ZEN was found to be more potent than its metabolite. ZEN even mediated greater growth stimulation than E2 with a significant increase of 1.22 fold of total cell protein compared to the solvent control. α-ZEL induced significant proliferation at 100 nM reaching 1.13 fold of total cell protein, compared to the control. No cytotoxic effects were observed for both *Fusarium* metabolites at the tested concentrations. AOH did not induce proliferation, but was found to be cytotoxic at the highest concentrations of 5 and 10 µM. Due to these contrary effects on cellular proliferation on the one hand, and cytotoxicity on the other hand, it was not reasonable to generate curve fits and, more importantly, not possible to calculate combinatory effects by the *combination index theorem* (Chou 2006).

**Fig. 5:**
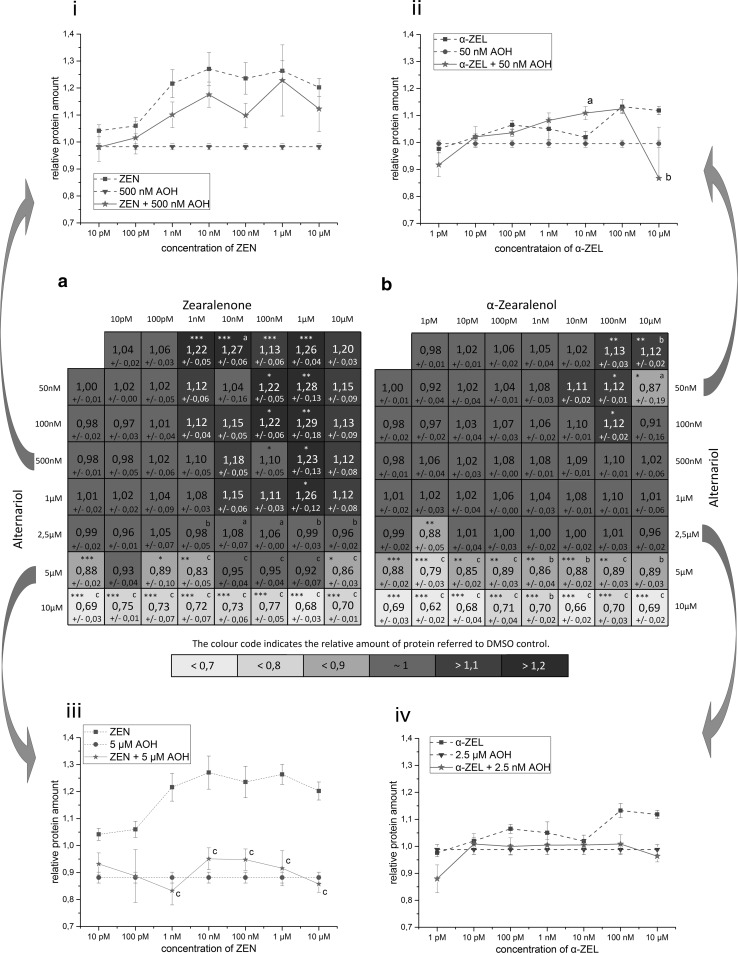
Heatmaps showing relative effects on cellular protein amount measured by the SRB assay, of single mycotoxins and binary combinations of AOH with (**a**) ZEN or (**b**) α-ZEL (mean, SEM). Protein amount of the solvent control was set to 1.* Four line graphs* covering whole concentration ranges of ZEN or α-ZEL in combination with one low (i, ii) and one high (iii, iv) concentration of AOH are extracted exemplary from the data set for comparison with the effects of single substances. Significant differences of effects to the solvent control are indicated by* asterisks*, significant differences of the binary mixtures to measurements with ZEN or α-ZEL, as single compounds, are indicated by a, b or c (*, ^#^ *p* > 0.05; **, ^##^ *p* > 0.01;***, ^###^ *p* > 0.001). All measurements were repeated in at least three independent biological replicates, each measured in technical triplicates

Figure 5a, b illustrates heatmaps of all tested combinations and respective data on relative cell protein related to the solvent control. In accordance with measurements of single compounds, combinations of moderate to high concentrations of ZEN (100 nM to 1 µM) and low concentrations of AOH (up to 1 µM) showed proliferative effects, whereas such effects could hardly be found in combinations with α-ZEL. Nevertheless, in both data sets for combinations at high concentrations of AOH (5 and 10 µM) predominantly cytotoxic effects were detected. In order to visualize the impact of AOH on the proliferative effects of ZEN and α-ZEL, four exemplary graphs were drawn out of the data set. For each *Fusarium* compound, two line graphs are plotted showing effects of combinations with AOH in low and high concentrations, respectively, as well as the effects of the single substances (Fig. 5i–iv).

## Discussion

### Evaluation of estrogenic effects

Measurements of single mycotoxins confirmed previous reports that α-ZEL is the most potent estrogen of the tested mycoestrogens followed by the parent compound ZEN and that AOH possesses comparably low estrogenic potency (Frizzell et al. 2011; Hagler et al. 1979; Lehmann et al. 2006; Metzler et al. 2010). Comparison of EC50 values in the AlP assay revealed a clear ranking according to estrogenicity as follows: α-ZEL > E2 > ZEN > AOH. However, these EC50 values depend on the maximum effect which can be achieved. This was found to be a substance specific property and explains why α-ZEL exerts a lower EC50 value than E2 but may, however, not be considered as a stronger estrogen. Visual interpretation of the data shown in Fig. 1b clearly results in a more reasonable ranking of the substances according to estrogenicity which is also reflected by the corresponding concentrations of substances giving 50 % of the effect of E2 shown in Table 1: E2 > α-ZEL > ZEN > AOH. Not only the concentrations at which the substances induced effects but also the maximum potency of AlP activation differed among the compounds (Fig. 1b). All tested concentrations of α-ZEL, ZEN or AOH showed significantly lower effects than E2. These findings suggest that α-ZEL, ZEN and AOH act as partial agonists in this estrogenic system.

Interestingly, measurements of total cell protein levels in the SRB assay did not reflect these findings with respect to cell proliferation. Dose–response evaluation in the SRB assay of ZEN, as single substance, revealed higher proliferative effects than those caused by α-ZEL or even by E2. This was not found in a classical E-screen study using MCF7 breast cancer cells (Molina–Molina et al. 2014). However, considering that an incubation time of 144 h was applied by Molina–Molina et. al., the strong proliferative effect of ZEN, found in our study, may depend on differences in the duration of exposure as well as cell line-specific responses.

In the present study, the maximum induction of AlP measured was not generated by the natural estrogen hormone E2. Several combinations of AOH with ZEN or α-ZEL induced higher levels of AlP activity than E2, although not statistically significant from the E2 effect (*p* < 0.05). The highest effect was found for the combination of 10 nM ZEN and 5 µM AOH. Co-incubation of highly potent combinations with the ER inhibitor ICI 182,780 indicated that these strong effects also depend on ER signaling. In line with the potency of the single compounds, combinations of AOH and α-ZEL showed increased AlP activity at much lower concentrations compared to combinations with ZEN. Combinations of α-ZEL and AOH in concentrations below 1 nM and 10 µM, respectively, resulted throughout in synergistic effects.

A general principle describes combinatory effects of substances that do not interact as simply additive. Accordingly, interactions between compounds are indicated, when the combinatory effect is not additive. The *combination index theorem* is the mathematical model of first choice, which allows quantitative assessment of synergistic or antagonistic effects (Chou 2006). To apply this model, effects of single substances and combinations need to be described by values between zero and one, standing for 0–100 % estrogenic stimulus. However, since α-ZEL, ZEN and AOH act as partial antagonists, 100 % of activation needed to be set to a common value. Therefore, the value of 3.4, which lies slightly above the highest measurements obtained during this study, was defined as 100 % AlP induction for calculation of the *combinatory index* (CI). Since only relative values are applied here, this approach is not expected to have a major impact on the calculation of *combinatory indices* and even minor effects on final conclusions, to be drawn from these. However, high concentrations of α-ZEL and ZEN, above 1 and 10 nM, respectively, were not included in the calculations, as these would falsify the CI calculations of the whole data set, due to the partial antagonistic characteristics of the substances. Therefore, CI calculation was limited to combinations with ZEN below 100 nM and α-ZEL below 10 nM. However, considering the fact that the single substances act as partial agonist and therefore do not reach the potency of E2, but several combinations do, this may be considered as synergism *per se*. This was demonstrated by statistical analysis, which revealed significant differences of effects, of all single mycotoxin concentrations to the effect of E2 (thus significantly lower than the AlP level reached by E2) that is no longer true for several combinations. Summing up these results, interactions between AOH and ZEN or α-ZEL were found to be synergistic over wide concentration ranges. A reasonable explanation for this synergism could be the fact that AOH has been reported to predominantly bind ER-β, whereas ZEN shows higher affinity to ER-α (Kuiper et al. 1998; Lehmann et al. 2006). This mechanism seems plausible since Ishikawa cells are known to express both estrogen receptor subtypes (Johnson et al. 2007) and are thus well-suited for the performed experiments.

Combinations with the two highest concentrations of AOH (2.5 and 5 µM) generally showed a decline of estrogenic effects compared to lower doses although still producing a significantly enhanced level of AlP activity. Here, cytotoxic effects of the mycotoxins presumably overlapped with estrogenic effects, as it was shown by the SRB measurements. The data of the SRB assay, in general, indicated that AOH reduces the proliferative effects of ZEN and α-ZEL in a dose dependent manner. In fact, at these high concentrations of AOH, of 2.5 and 5 µM, combinations showed slightly cytotoxic effects, which, however, are more pronounced in the presence of α-ZEL than of ZEN. This reflects the results of cell protein measurements with the single substances, which showed that ZEN possesses a stronger proliferative potential than α-ZEL under these experimental conditions and, therefore, may counteract the cytotoxicity of AOH more effectively. Interestingly, the reduction of protein amount is not explicitly reflected by a dramatic decrease of induction of AlP activity. The fact that the estrogenic effects on AlP are in general much more pronounced than the proliferative effects may give a reasonable explanation for this. An experimental setting with 48 h of incubation duration may simply be too short to observe stronger effects in proliferation in a cell line with a doubling time of 27–36 h (Nishida 2002).

Measurements on constant ratio combinations between ZEN or α-ZEL and AOH also revealed, according to the CI evaluation, mainly synergistic effects. However, evaluated concentrations of the combinations giving 50 % of the effect of E2 did not reveal a shift of the dose–response curve α-ZEL when combined with AOH. A slight shift to the left could be detected for the curve of ZEN. ZEN alone reaches 50 % effect of E2 at 1.423 nM, whereas in combination with AOH (1:250) this effect is reached at 865 pM (+216 nM AOH). Evaluation of combinatory effects resulted in quite extreme CI values (Fig. 4c, d), especially in low effect ranges considering that the data from the dose–response curves are reflected (Fig. 4a). It may be considered, however, that due to the 1:250 ratio several of the combined AOH concentrations are far below the effect range of AOH (below 50 nM). Accordingly, only slight changes in the low effect ranges might result in calculations of very strong combinatory effects. Anyhow, for both combinations, the evaluated dose–response curves again reveal similar effects to what was found previously. The maximum effects of the combinations exceed those of ZEN and α-ZEL and reach the effect level of E2. As these effects were found to depend on ER signaling, they may be considered as estrogenic synergistic effects *per se*.

### Potential relevance for risk assessment on mixtures of mycoestrogens

This study revealed synergistic estrogenic effects of the mycotoxin AOH in combination with ZEN or its phase I metabolite α-ZEL. Whether these combinations may bear endocrine disruptive potential, as it is already known for ZEN, still needs to be answered by detailed elucidation of potential adverse effects in vivo. However, since mycotoxins are natural food contaminants, co-exposure of the human population seems to be most likely in many settings. As a consequence, priority shall be attributed to combinations of mycoestrogens and estrogenic food constituents in general. Recently, considerations on combinatory effects initiated a paradigm shift in risk assessment of all kinds of chemical substances that stipulates mixture effects to be included in future (EFSA 2013). This applies to substances with all possible modes of action; however, it seems to be of special importance for endocrine disruptors. Due to the high sensitivity of endocrine systems in general, potential effects of compounds might be pronounced even at very low concentrations. There are some studies investigating the combinatory effects of prominent anthropogenic endocrine disruptors like bisphenol-A, phthalates, polychlorinated biphenyls (PCBs) and dioxins, which indicate that endocrine effects of mixtures possibly occur below NOELs (Couleau et al. 2015; Crofton et al. 2005; Li et al. 2012b). Considering co-contamination of food products by several substances, human exposure to mixtures seems to be even more likely. Our data suggest that also mycoestrogen mixtures may lead to adverse effects even at very low concentrations due to synergistic interactions. As a consequence, current limit values like the tolerable daily intake (TDI) for humans or maximum tolerated levels for mycotoxins in food may need reconsideration, as they are at present still derived from risk evaluation of single substances (EFSA 2011). Our study highlights the importance of combinatory effects to be incorporated in future approaches of risk assessment.

## Abbreviations

AOH: Alternariol
ZEN: Zearalenone
α-ZEL: α-Zearalenol
E2: 17β-Estradiol
PBS: Phosphate-buffered saline
DMSO: Dimethyl sulfoxide
TCA: Trichloroacetic acid
SRB: Sulforhodamine B
CI: Combination index
SEM: Standard error of the mean
AlP: Alkaline phosphatase
LOEL: Lowest observed effect level
NOEL: No observed effect level
